# Canker Development and Biocontrol Potential of CHV-1 Infected English Isolates of *Cryphonectria parasitica* Is Dependent on the Virus Concentration and the Compatibility of the Fungal Inoculums

**DOI:** 10.3390/v14122678

**Published:** 2022-11-29

**Authors:** Pedro Romon-Ochoa, Jack Forster, Ruth Chitty, Caroline Gorton, Alex Lewis, Amy Eacock, Quirin Kupper, Daniel Rigling, Ana Pérez-Sierra

**Affiliations:** 1 Forest Research, Tree Health Diagnostics and Advisory Service (THDAS), Alice Holt Lodge, Wrecclesham GU104LH, UK; 2 Swiss Federal Institute for Forest, Snow and Landscape Research WSL, Zuercherstrasse 111, 8903 Birmensdorf, Switzerland

**Keywords:** Cryphonectria hypovirus 1, England, transmissions, preservations, seedlings, branches, concentration, compatibility, real-time PCR

## Abstract

Biological control of *Cryphonectria parasitica* fungus, causal agent of chestnut blight, by virus infection (hypovirulence) has been shown to be an effective control strategy against chestnut blight in Europe and some parts of North America. The most studied mycovirus is the Cryphonectria hypovirus 1 (CHV-1) type species of the *Hypoviridae* family. To efficiently provide biocontrol, the virus must be able to induce hypovirulence in its fungal host in chestnut trees. Here, two different CHV-1 subtype I virus strains (E-5 and L-18), gained by transmissions, were tested for their hypovirulence induction, biocontrol potential, and transmission between vegetatively compatible (VCG) and incompatible fungal isolate groups in sweet chestnut seedlings and branches. Both strains of CHV-1 showed great biocontrol potential and could protect trees by efficiently transmitting CHV-1 by hyphal anastomosis between fungal isolates of the same VCG and converting virulent to hypovirulent cankers. The hypovirulent effect was positively correlated with the virus concentration, tested by four different reverse-transcription PCRs, two end-point and two real-time methods, one of which represents a newly developed real-time PCR for the detection and quantification of CHV-1.

## 1. Introduction

Chestnut blight is a disease of the *Castanea* species caused by the ascomycete *Cryphonectria parasitica* (Murrill) M. E. Barr. The fungus originates from Eastern Asia [1] but it has caused severe epidemics resulting in death and dieback of both *Castanea dentata* (Marshall) Borkh. in North America where it was introduced in the late nineteenth century [2], and on sweet chestnut (*Castanea sativa* Mill.) in most of continental Europe, where it was first introduced in Italy in 1938 [3]. Diseased chestnut trees exhibit crown dieback above girdling cankers on the trunk and/or branches and profuse epicormic growth may be observed below the cankers. Additional signs include orange fruiting bodies that erupt through swollen lenticels and/or whitish mycelial fans that form beneath the bark and spread into the phloem and cambium tissue. Planting stock, timber, bark, and seeds are pathways of introduction and long-distance dispersal.

England was considered free of chestnut blight until 2011, when *C. parasitica* infections were discovered on 90 young saplings of sweet chestnut planted in a nursery farm in Warwickshire [4]. They originated from the same nursery in Europe; the saplings were imported and planted in 2007 and some trees died and were replaced in 2010. This stimulated surveys between 2011 and 2012 where the fungus was identified on recently planted saplings at a further eight orchard sites located in Devon, Herefordshire, Kent, Norfolk, Somerset, and Sussex. All affected trees were eliminated. In 2013, United Kingdom introduced tighter import controls, meaning that the movement of sweet chestnut trees in, around and out of England needed to be accompanied by official documentation confirming that they were from an area free of the disease [5]. In August 2016, chestnut blight was confirmed on a recently planted tree in Kent that was removed. Until 2016, all findings of the disease in the UK were exclusively in orchards or recently planted individual young trees, and therefore their eradication was relatively easy. However, in December 2016, *C. parasitica* was isolated from four mature trees growing in a car park in Devon. Additional Forestry Commission of England and Animal and Plant Health Agency surveys were initiated, and *C. parasitica* was subsequently diagnosed in a woodland about 1 km away from the last infected site. A trace-forward and trace-back exercise was initiated which revealed multiple positive findings in England between 2017 and 2020 [6,7].

Cryphonectria hypovirus 1 (CHV-1) is the type of species of the family *Hypoviridae* [8]. Hypoviruses are RNA viruses located in the cytoplasm membrane vesicles of their fungal hosts, without a coat protein, and with double-stranded RNA (dsRNA) replication form [9]. Its mode of transmission is through hyphal anastomosis that can be formed between fungal individuals or through conidia to asexual offspring. When individuals of the fungus belong to the same vegetative compatibility group (VCG), hyphal fusions can occur between them, which provides an opportunity for the transmission of the hypovirus [10]. When isolates are incompatible, hyphal fusion is unlikely and transmission of the hypovirus will not occur or occurs to a lesser extent [11]. Cryphonectria hypovirus 1 acts as a biocontrol agent of sweet chestnut blight in Europe and some parts of North America (Virginia, Wisconsin, Maryland), where it has been released, because it causes reduced growth, pigmentation, sporulation, and virulence on its fungal host [12]. In England, this virus was detected for the first time in November 2017 [6], and since then it has been observed in a small proportion (seventeen isolates out of 350), and at low concentration (ranging between 1.9 and 48.1 ng/µL of RNA extract after reverse-transcription PCR (RT-PCR), equivalent to approximately 4.3–110.1 ng/mg of mycelium) [6,7,13].

As CHV-1 has both low incidence and low concentration in *C. parasitica* isolates in England, but high potential to be used as a biocontrol agent, the objectives of this study were to: (1) transmit the hypovirus to English isolates of EU-10 (dominant in London) and EU-9 (dominant in Devon) VCGs from already infected isolates from Europe of the same or proximate VC groups; (2) preserve those infected English isolates and compare their viral concentration before and after preservation; (3) assay the ability of the virus to control the canker development in plant material under controlled conditions (seedlings and branches); and (4) describe a rapid detection and novel quantification method for this mycovirus.

## 2. Material and Methods

### 2.1. Viral and Fungal Strains

Viral and fungal strains used in the current study are shown in Table 1. Two hypovirus strains were tested: CHV1-M2273 haplotype E-5, and CHV1-M2357 haplotype L-18 (designation of sequence haplotypes according to Gobbin et al. 2003). Both virus strains belong to CHV1 subtype I [14] and were originally found in *C. parasitica* isolates from southern Switzerland. The hypoviruses were first transmitted to the English fungal isolates SDA540 (vc type EU-10) and WAP125 (vc type EU-9) (Table 1), which were subsequently used as virus donors for transmission into the virus-free English fungal isolates FTC687, WAP706, and POWP709. These fungal strains were originally isolated by placing small pieces of bark lesions onto malt agar plus streptomycin (MA + S), and then culturing them on potato dextrose agar (PDA). Virus transmission was accomplished through hyphal anastomosis by the coculture of virus-infected donor and recipient fungal strains on a PDA plate (9 cm in diameter), as described previously [15].

On the other hand, four virus-free English fungal isolates were used. LAP731 and FTC687 VIRUS-FREE, both isolated in 2021 from London, WAR706 VIRUS-FREE from Devon, and DIG460 isolated in 2020 from Devon [7], belonging to the VCGs EU-10, EU-10, EU-9, and EU-9, respectively, were used as virulent fungal isolates.

### 2.2. Preservation of Virus-Infected Fungal Strains and Assessment of Their Viral Load

Each virus-infected fungal isolate was preserved after growing on both PDA plates and PDA plates with two sterile filter disks (Whatman 1820-055), square portions of 1 × 2 cm at 25 °C under a 16 h photoperiod of 2500 lux for 14 days. Initially, six mycelial plugs with abundant sporulation were deposited at −80 °C in cryovials with 800 µL glycerol 22%, previously heavily vortexed, and immediately frozen in liquid nitrogen just before entering the ultra-freezer within a mapped cryogenic box.

The filter disk portions were transferred to a plastic box with silica gel at −20 °C in the form of colonized and dried (48 h under laminar flow chamber) filter disk portions included within sterile paper envelopes.

To quantify the virus content of each isolate, before and after two months of the two types of preservation (two replicates), incubation, RT-PCR, and electrophoresis methods were used as previously described in Pérez-Sierra et al. [6].

### 2.3. Inoculation of Sweet Chestnut Seedlings and Branch Segments, and Fungal Re-Isolation

Eighteen-month-old chestnut tree seedlings (*C. sativa*; provenance, Delamere, United Kingdom) of approximately 1.3 m high and approximately 2.5 cm wide (diameter) at the bole, were purchased from an English nursery free of the disease (Delamere) and grown outside for one year from May 2021 to May 2022. They were moved to a biosafety level 3 greenhouse at Forest Research Holt quarantine laboratory and acclimated to the greenhouse environment for a week (BSL3) which was temperature-controlled (25/20 °C, day/night), with a photoperiod of 8 h of light.

Two different assays (I and II) were done. Both assays were repeated by using sweet chestnut branch segments 25 cm long and about 2 to 2.5 cm in diameter previously kept one week in a cold store. Both plant materials were supplied with tap water twice a week. All inoculations were done using mycelial plugs taken from actively growing cultures after seven days on PDA. The viral content in the hypovirulent isolates was re-checked following Pérez-Sierra et al. [6] at that point.

For assay I (individual inoculations to investigate virulence), the six different virus-infected *C. parasitica* strains were inoculated onto seedlings and branch segments. In the case of the seedlings, four biological replicates were used for each strain and for the virus-free controls plus PDA controls. Thus, a total of 44 seedling lesion areas were measured after approximately two months. In the case of the branch segments, three branch segments were randomly held in buckets distributed across a mapped trial with 11 buckets. After inoculation, the holes were sealed with LacBalsam (Compo, Eggenfelden, Germany) to prevent desiccation. At the time of harvesting, (53 days after the individual inoculations), the length and width of the cankers were measured, and the canker area was calculated using the ellipse formula, A = L/2 × W/2 × pi, where area equals half-length per half-width per pi number.

For assay II (challenge inoculations to estimate biocontrol potential), a total of 64 seedlings (only VCG compatible combinations) or 140 branch segments (all the combinations) were used. Two weeks following primary inoculations with the virulent (virus-free) fungal strains, challenge inoculations were made with the virus-infected strains or PDA. Eight inoculations regularly distributed along the periphery of a virulent canker were carried out [16]. After inoculation, the holes were sealed with sterile water-soaked sterile cotton, parafilm and aluminium foil to prevent desiccation. At the time of challenge inoculation, the length and width of the original cankers were measured, and the canker area was calculated using the ellipse formula. Canker expansion after the biocontrol treatments was also measured after 53 days after the challenges by using the same approach. In the case of the chestnut branch segments, five branch segments were randomly held in buckets distributed across a mapped trial with 28 buckets.

All cankers were sampled at the end of the experiment to verify virus infection. Four bark samples (top, two middles, and bottom of the canker) were taken from each canker using a bone marrow biopsy needle (diameter, 1.6 mm; Microlance 3; BD, Huesca, Spain). Bark plugs were placed on malt agar plates containing streptomycin at a concentration of 40 mg/L and incubated at 20 °C for four days. The outgrowing mycelium was transferred onto PDA (39 g/L, BD Difco) plates, which were then incubated at 25 °C in the dark for 7 days.

### 2.4. Direct One-Step Reverse Transcription PCR and Comparison with Other Endpoint and Real-Time Virus Detection Methods

The simplified and reliable one-step RT-PCR technique developed by Urayama et al. [17] for virus detection in *Magnaporthe oryzae* without nucleic acid extraction, more importantly already tested for *C. parasitica* viruses by the same study and the ones of Aulia et al. [18] and Suzuki et al. [16], was employed to detect the virus in the fungal isolates re-isolated from cankers. This method entails stabbing the central 7 days growing region of mycelial colony on PDA with a toothpick and dipping the toothpick into a 19 µL premixed RT-PCR mixture prepared according to the protocol for PrimeScript One Step RT-PCR version 2 (Dye Plus) (TaKaRa Bio, Saint-Germain-en-Laye, France). 

PCRs were performed using 19 µL reaction mixtures containing 10 µL of 2 × Dye plus buffer, 0.4 µL of each forward HVEP1 (5′-TGACACGGAAGCTGAGTGTC-3′) and reverse HVEP2 (5′-AGCGCGAATTTCTTGTCG-3′) primers (20 mM) [14], 0.8 µL of PrimeScript one step enzyme mix, 7.40 µL RNase free water per reaction, and RNA sample by toothpick dipping. Thermal cycling profiles were 50 °C for 30 min; 95 °C for 2 min; 40 cycles at 95 °C for 30 s, 53 °C for 30 s, and 72 °C for 60 s. PCR products were visualized under UV illumination on a 1% agarose gel, made with 80 mL of 0.5 × TBE buffer, stained with 5 µL of GelRed (Merck, Gilligham, UK), and run at 90 V for 45 min in a Wide Mini-Sub Cell GT Electrophoresis System (Bio-Rad, Watford, UK). The approximately 394 bp band was quantified in comparison to a CSL-MDNA-1 kb ladder (Cleaver Scientific, Warwick, UK).

This detection method, therein after called the End-point TaKaRa II Toothpick, was compared with that described in Pérez-Sierra et al. [6], comprising RNA extractions with on-column DNA digestions and named End-point Qiagen Extract, and with a new method, called Real-time TaKaRa III, either from RNA extracts or colony toothpick. This method was used for the first time in the present study and represents, to the best of our knowledge, the first time that a RT-qPCR was designed for CHV-1. It implies the use of a One Step PrimeScript III RT-qPCR kit (TaKaRa Bio, Saint-Germain-en-Laye, France) in combination with the primers and probes indicated in Table 2, designed by using OligoArchitect^TM^ Online (Sigma-Aldrich, Gillingham, UK). Real-time PCRs (qPCRs) were carried out on a LightCycler 480 (Roche, Welwyn, UK). For the assay, 0.4 µM of the primers and probes were used in a 20 µL reaction volume comprising 10 µL 2 × One Step PrimeScript III RT-qPCR Mix, 0.4 µL of each primer and probe, 6.6 µL RNase free water, and 1 µL of RNA sample (or toothpick dipping) per reaction. Each sample was performed in triplicate. Thermal cycling conditions were 50 °C for 30 min and 95 °C for 50 s, followed by 40 cycles of 95 °C for 25 s and 53 °C for 1 min. Fluorescent detection occurred at the end of each 53 °C step. The cycle threshold (Ct) value was calculated automatically using the LightCycler software (Roche, Welwyn, UK) with absolute quantification using a second derivative maximum setting with 465–510 and 533–580 nm channel filters for analysing the specific amplicons and the actin internal control.

**Figure 1:**
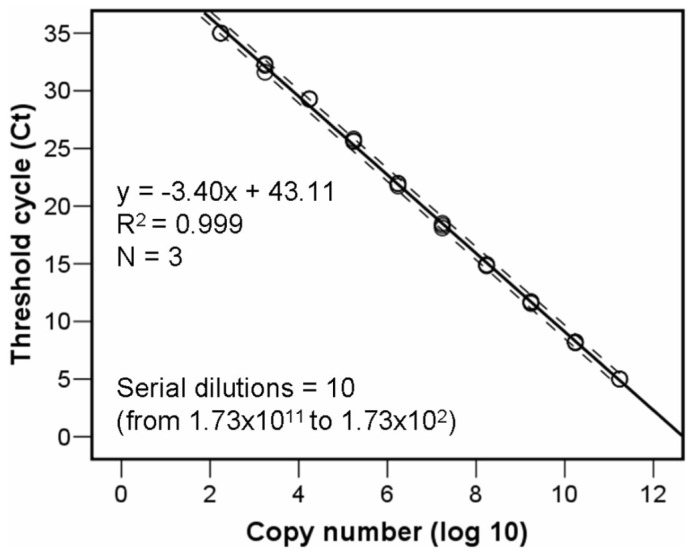
Regression equation relating the CHV-1 virus copy number with threshold cycle values.

A ten 1:10 points serial dilution of the 94 bp specific qPCR product synthesized de novo and cloned into a plasmid pUC-GW-Kan (Azenta Life Sciences, Leipzig, Germany), and posterior regression equation analyses, permitted estimating the fragment copy number, actual number of the CHV-1 virus, in real samples depending on the mean threshold cycles number per triplicate wells (Figure 1). For calculating the copy number in the original plasmid aliquot, the copy number was calculated using the following equation: number of copies/µL = [(6 × 10^23^) × (DNA concentration, 500 ng/µL)/molecular weight of one plasmid], where 6 × 10^23^ is the number of copies per mole, DNA concentration is given in grams per microliter, and the molecular weight of one plasmid is in grams per mole assuming a plasmid size of 2627 bp and a 1 bp molecular weight of 660 g/mole.

### 2.5. Statistical Analyses

Analyses were conducted in R (version 4.1.0), with graphics produced using ggplot2 in R [19,20,21,22]. In all, [23] linear mixed effects models were applied to the data using the square root internal lesion area as the response (to meet normality assumptions) and random effects for isolate. The interaction of row and column position (numeric) was included in all initial models as fixed effects. Akaike Information Criteria (AIC) were used to select the most efficient model. Having selected the most efficient model, an Analysis of Variance (ANOVA) (Type 2 F tests, [24]) was used to determine the significance of fixed effects, with non-significant effects removed from the final model. Estimated marginal means with pairwise contrasts (Tukey’s HSD corrections, 22) were used to show significant differences within fixed effects.

Considering assay I with seedlings, three initial models were fitted to the data using different virus fixed effects: (1) virus strain, (2) virus absence/presence, and (3) original virus concentration.

Regarding assay I, using branches, the same three initial models were fitted to the data.

Considering assay II with seedlings, three separate models were applied to the data, one including the interaction of primary and challenge inoculation, one with the interaction between the primary inoculation and challenge inoculation strain, and one with the interaction between the primary inoculation and control/virus, with all models including row and column position (numeric) and initial lesion area (all challenge inoculations were compatible, so this effect was excluded) as additional predictors.

Regarding assay II using branches, the data indicated that most samples had reached the maximum achievable lesion size by the end of the experiment, therefore data were analysed in two ways: (1) using the binary response of not achieved/achieved maximum lesion size, and (2) the analysis of actual lesion size for the subset of samples that had not achieved maximum lesion size. The number of samples achieving maximum lesion size versus not was used as a two-column matrix response in a generalized linear model with binomial errors and a logit link function. Two separate models were applied to the data, one including the interaction of primary and challenge inoculation and one with the interaction between primary inoculation and challenge inoculation strain, with both models including challenge inoculation compatibility as an additional predictor. AIC values were used to determine the most efficient model, with the analysis of deviance (likelihood ratio chi-square tests, [25]) used to determine significant effects.

## 3. Results

### 3.1. Transmissions

All the co-culture hypovirus transmissions were successful, with gained virus concentrations ranging between 234.37 and 612.83 ng/µL of RNA extract after the virus-specific RT-PCR [6], equivalent to about 536.7–1403.3 ng/mg of mycelium. This represents a mean increment of 912.8 ng of amplicon per mg of mycelium with respect to the determined concentrations in the wider environment.

### 3.2. Preservations

Both glycerol and the paper disk methods worked nicely in general to preserve the CHV-1 strains in the infected fungal isolates, and although there were no significant differences, the glycerol method results were on average slightly better than the paper disks preservation method (Table 3).

### 3.3. Assay I, Pathogenicity Test

**Figure 2:**
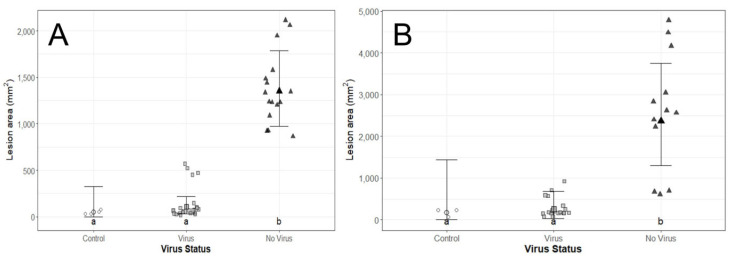
Lesion area produced by virus-infected and virus-free *C. parasitica* isolates. Points with error bars represent estimated marginal means with 95% confidence intervals. Lettering indicates significant differences by treatment. (**A**) Seedlings. (**B**) Branch segments. See Material and Methods third paragraph for experimental details.

**Figure 3:**
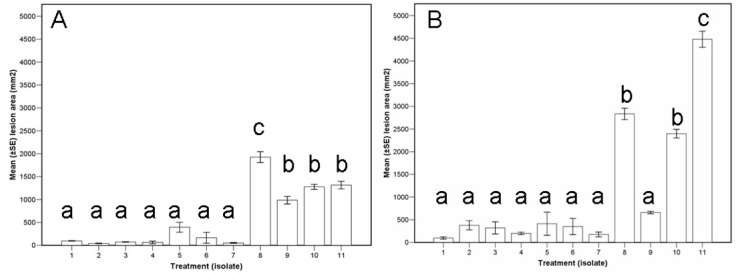
Lesion area 53 days after individual inoculation (assay I) with ten isolates of Cryphonectria parasitica (1–3 infected with the hypovirus CHV1-M2273, 4–6 infected with CHV1-M2357, and 8–11 virus-free isolates) and a PDA control (number 7). (Eleven treatments). Bars with the same letter are not significantly different based on Tukey′s test. (**A**) Seedlings (*n* = 44). (**B**) Branch segments (*n* = 33). See Material and Methods third paragraph for experimental details.

Using seedlings, initial correlations suggested strong (>0.7) positive correlation between all pre- and post-experimental viral concentrations. The most efficient model included the absence/presence of the virus only (not strain or viral concentration; (F_2,8_ = 40.1, *p* < 0.0001). Lesions caused by virus-free isolates were significantly larger than those caused by virus-infected isolates (Figure 2A and Figure 3A). Lesion areas of virus-infected isolates were not significantly larger than the control inoculations with PDA (Figure 2A). There was a notable variation across fungal isolates, with the LAP731 (virus-free) isolate having markedly larger lesion areas than the other virus-free isolates (Figure 3A). The results showed a negative correlation between the lesion area and the original or post-harvesting viral concentration (Supplementary Table S1). This is consistent with that as the viral concentration is higher, the lesion area is smaller. Besides, the original viral concentration was positively correlated with all post-harvesting viral concentrations, except virus copy number (Supplementary Table S1). Strain L-18 was correlated with higher virus concentrations, even virus copy number, and thus with smaller lesion areas (Supplementary Table S1).

Similarly, the most efficient model using branch segments also included the absence/presence of the virus only (not strain or viral concentration; (F_2,8_ = 11.8, *p* = 0.004). Lesions caused by virus-free isolates were significantly larger than those with the virus or negative controls (Figure 2B). Lesion areas of those fungal isolates with the virus were not significantly larger than the controls (Figure 2B and Figure 3B). There was notable variation across fungal isolates, with the FTC687 (virus-free) isolate having markedly smaller lesion areas than the other virus-free isolates (Figure 3B). Similar correlations were found than those using seedlings (Supplementary Table S2).

### 3.4. Assay II, Biocontrol Potential

**Figure 4:**
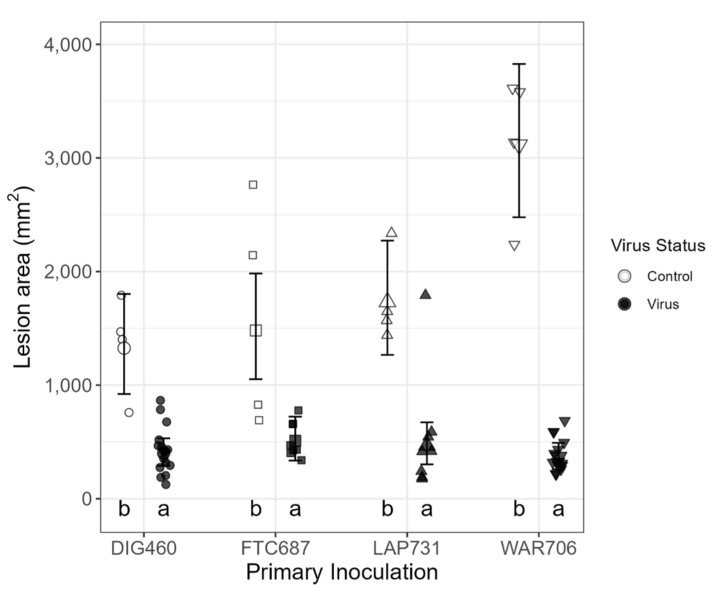
Lesion area by primary inoculation and challenge inoculation with virus or without virus (control) using seedlings. Points with error bars represent estimated marginal means with 95% confidence intervals. Lettering indicates significant differences between challenge inoculation with virus compared to the control. See Material and Methods third paragraph for experimental details.

Using seedlings, the most efficient model included the challenge inoculation virus absence/presence only. There was a significant interaction between virus absence/presence and primary inoculation isolate; (F [3,26] = 8.21, *p* = 0.0001) along with a main effect of virus absence/presence (F [1,56] = 165, *p* <0.0001). Challenge inoculation virus presence was associated with significantly smaller lesions (Figure 4). The results showed a negative correlation between the lesion area and the original or post-harvesting viral concentration (Table 4). The original viral concentration was positively correlated with all post-harvesting viral concentrations, including virus copy number. Strain L-18 was correlated with bigger virus concentrations and thus with smaller lesion areas (Table 4).

Using branches, all successful challenge inoculations were compatible, so this effect was excluded in the analyses. Thus, the most efficient model included challenge inoculation compatibility only; (LR chi-sq = 34.1, *p* < 0.0001). Those treatments where challenge inoculations were compatible were significantly less likely to achieve maximum lesion area versus incompatible and control challenge inoculations. The second and third most efficient models included challenge inoculation viral strain; (F_1,13_ = 7.7, *p* = 0.016) along with primary inoculation isolate (F_3,13_ = 4.6, *p* = 0.021). Challenge inoculation strain 2 (L-18) was associated with smaller lesions. Smaller lesion areas were related with higher post-harvest virus concentrations (Table 5).

### 3.5. New Real-Time PCR

Real-time TakaRa III Toothpick method data revealed that this new PCR will be very useful and faster for detecting the CHV-1 virus in more and even less concentrated (common in England) cultures because those results indicate around four-fold times more amplification efficiency than the protocol normally used (End-point Qiagen Extract) and without the need to perform either RNA extractions or gel electrophoresis. 

**Figure 5:**
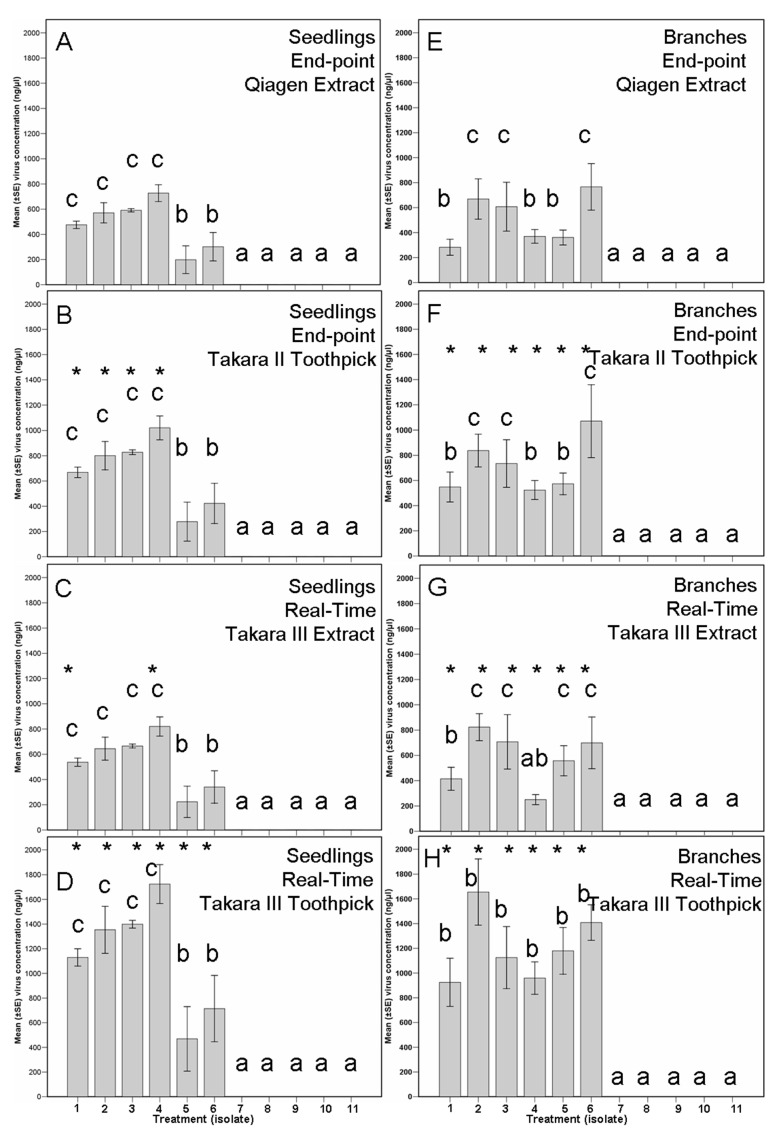
Virus concentration within the re-isolations after 53 days post individual inoculation (assay I, (**A**–**D**) using seedings, (**E**–**H**) using branch segments) with ten isolates of *Cryphonectria parasitica* (1–3 infected with the hypovirus CHV1-M2273, 4–6 infected with CHV1-M2357, and 8–11 virus-free isolates) and a PDA control (number 7). (Eleven treatments). Bars with the same letter are not significantly different based on a Tukey′s test. (**A**) Seedlings (*n* = 44). (**B**) Branch segments (*n* = 33). Bars with an asterisk indicate significant differences against the same isolate treatment analysed using the Qiagen Extract End-point PCR method for the CHV-1 mycovirus.

The triplicate tests’ standard deviation was very low, giving very similar results per sample. Furthermore, the use of the ten 1:10 serial dilution of the cloned qPCR product permitted determining the detection limit of this PCR protocol in two viruses. This real-time PCR was tested against five different subtypes of the mycovirus (I, F1, E, D, and G) with positive results. However, the unique weak point of this method is that it could be more difficult to discriminate statistically significant differences among the different infected isolates in comparison with the other three methods, especially the end-point methods (Figure 5).

## 4. Discussion

Cryphonectria hypovirus 1 is known to induce hypovirulence in *C. parasitica* by reducing pathogenic growth and sporulation, hence the virus is used in Europe for disease control [12]. The virus has been introduced into continental Europe on multiple occasions in association with *C. parasitica* from countries such as Japan, China [1,26] and Korea [27], which are known to be the geographical origin of the fungus. Since those introductions, both *C. parasitica* and CHV-1 have spread widely [27]. Originally, such introductions are most likely to have occurred through the plant trade and/or the importation of Asiatic planting stock often intended for use in breeding resistance programs to chestnut ink disease caused by *Phytophthora cinnamomi* and *P. cambivora*. Six genetically distinct CHV-1 subtypes have been identified in Europe (I, D, E, F1, F2 and G) [14,28]. Subtype I (also known as the Italian subtype), and which is the only subtype that has been detected in England [6,7,13], is the most widespread, because it is commonly associated with mild hypovirulence. It is dominant in Italy, Switzerland, south-eastern France, Greece [29], Bosnia [30], Croatia [31], Slovenia [32], Macedonia [33], Turkey [34], and now also England [6,7,13], where the haplotype exactly matches haplotype E-5. The European distribution of the E-5 haplotype has not been well studied. It was described as a rare haplotype by Gobbin et al. [14], and it has been introduced in an experimental site near Monthey (Switzerland), where it became widely established [35]. The other subtypes, especially F1 with the type member (CHV1-EP713), are usually less abundant because the increased aggressiveness of the virus causes a very relevant reduction in fungal growth and therefore makes infected individuals less likely to persist. F1 and other subtypes have been found in some parts of France [36], Spain [37], eastern Turkey [34], Georgia [28], and Germany [38]. The CHV-1 virus does not occur naturally in the USA but is present in a few locations such as in Virginia [39,40], Wisconsin [41] or Maryland [42] where it has been released for the biological control of American chestnut blight. In Europe, several genetic re-combinations have contributed to the evolution of CHV-1 [28,43]. 

There have been relatively few studies on the impact of CHV-1 induced hypovirulence using plant material [39,44,45]. To the best of our knowledge, this is one of the first times that the pathogenicity and biocontrol potential of virulent and hypovirulent isolates of *C. parasitica* have been evaluated on both chestnut seedlings and cut branches [13,16], although other studies have used only cut branches [16,45], naturally infected trees [39,42], or both [44].

Our findings showed that there is a considerable difference in mean lesion area between virus-free isolates and those harbouring a high concentration of the CHV-1 virus, both when using seedlings and branches. The consistency of the plant material and controlled conditions used in our four assays gave high confidence about our observations and the conclusions that could be drawn from the data in relation to the efficacy of those artificially (by co-culture) infected English isolates.

Following findings of chestnut blight in the wider environment in England from late 2016 onwards [6], CHV-1 was first detected in *C. parasitica* populations at the end of 2017 but does not appear to be widespread based on the very low frequency of findings and the low concentration of detectable virus in affected isolates [6,7,13]. The results from this study suggest that the isolates FTC687 and WAR706 infected with the virus strain E-5 have the most potential for development as biocontrol agents of *C. parasitica* in the UK. These fungal isolates, which are now virus infected, initially originate from the east London area and Devon, where 77 and 60% of the total isolates belong to single VC groups (EU-10 in London and EU-9 in Devon) [6,7]. These outbreak clusters are possibly longstanding. The prevalence of a single VCG in each geographic area is likely to facilitate the spread of the virus and consequently hypovirulence [46,47]. It also opens the possibility of further evaluation of the field effectiveness of these two hypovirulent isolates in London and Devon FTC and WAR sites as pilot sites. However, further field experiments are needed before escalating the field inoculations by obtaining other VCGs transmissions for other locations where outbreaks have been detected with a low VCG diversity. Those will be comprised of periodic assessments of the treated and untreated cankers in the field every 6 months for a period of two years to determine their efficacy to control chestnut blight in the wider environment.

## 5. Conclusions

(1). Two CHV1 strains originating from Switzerland were successfully transferred by hyphal anastomosis to British isolates of *Cryphonectria parasitica* belonging to vc type EU10 and EU9, the predominant vc type in London and Devon, respectively.

(2). The biocontrol potential of the CHV1-infected British isolates was experimentally verified and found to be dependent on the inoculum compatibility and on the virus concentration, on both seedlings and cut branches.

(3). Real-time TakaRa III Toothpick method data revealed that this new PCR will be faster (no need for RNA extractions or electrophoresis), and more sensitive (around four times) for detection and quantification of the CHV-1 virus.

(4). Following a completed Pest Risk Assessment (PRA) and regulatory approval, the CHV1-infected isolates characterized in this study will be available for further biocontrol testing under field conditions.

## Figures and Tables

**Table 1:** Virus-infected and virus-free *Cryphonectria parasitica* strains used in this study.

Treatment Number	Fungal Strain	Description	VCG	Mating Type	Virus Strain
1	FTC687	Virus-infected strain, transmitted from SDA540 M2273	EU10 (2122-11)	MAT-2	E-5
2	WAR706	Virus-infected strain, transmitted from WAP125 M2273	EU9 (2111-11)	MAT-2	E-5
3	POWP709	Virus-infected strain, transmitted from WAP125 M2273	EU9 (2111-11)	MAT-2	E-5
4	FTC687	Virus-infected strain, transmitted from SDA540 M2357	EU10 (2122-11)	MAT-2	L-18
5	WAR706	Virus-infected strain, transmitted from WAP125 M2357	EU9 (2111-11)	MAT-2	L-18
6	POWP709	Virus-infected strain, transmitted from WAP125 M2357	EU9 (2111-11)	MAT-2	L-18
7	PDA CONTROL	Not Applicable (N/A)	N/A	N/A	N/A
8	LAP731	Standard virus-free strain	EU10 (2122-11)	MAT-2	N/A
9	FTC687 VIRUS-FREE	Standard virus-free strain	EU10 (2122-11)	MAT-2	N/A
10	WAR706 VIRUS-FREE	Standard virus-free strain	EU9 (2111-11)	MAT-2	N/A
11	DIG460	Standard virus-free strain	EU9 (2111-11)	MAT-2	N/A

**Table 2:** Oligonucleotides and fluorescent TaqMan probes used in this study for the real-time detection of the CHV-1 virus.

PROBE SPECIFIC FOR CHV-1	Tm * °C	GC %	ΔG Kcal/mol
CHV1-F: 5′-TGAGGAACGTCAACTTCG-3′	53.8	50.0	23.2
CHV1-R: 5′-TTGTGACGACGGAAATAATC-3′	54.3	40.0	24.10
HVEP1 Fluo: 5′-56-FAM/TGACACGGAAGCTGAGTGTC/3BHQ1/-3′	60.5	55.0	26.70
**PROBE FOR INTERNAL CONTROL TARGETING ACTIN mRNA & DNA**			
CpActinCF1: 5′-CCATGGTATCATGATTGGTATG-3′	58.4	41	25.0
CpActinCR1: 5′-TACCGCAGAGTCAGGATA-3′	53.8	50	22.4
CpActinCP1: 5′-56-JOE/TCATCACCAACATACGAGTCCTTCTG/3BHQ1/-3′	66.2	46	33.6

* Tm, melting temperature (salt adjusted); GC %, percentages of GC base pairs; ΔG, thermodynamic entropy at 1M NaCl at 25 °C, pH 7.

**Table 3:** Viral concentrations (ng/µL) before and after two months of two types of preservations (see methods second epigraph).

Treatment Number	Strain	Before Preservation	After Glycerol Preservation	After Disks Preservation
1	FTC687	234.37	293.60 (±6.30) a	240.30 (±140.96) a
2	WAR706	407.09	371.84 (±47.94) a	240.98 (±295.33) a
3	POWP709	371.42	364.70 (±44.69) a	230.96 (±314.99) a
4	FTC687	612.83	382.92 (±47.33) a	236.83 (±199.30) a
5	WAR706	458.04	425.86 (±99.05) a	137.64 (±185.07) a
6	POWP709	343.02	412,37 (±75.27) a	144.00 (±195.52) a
Mean total		404.46	375.21 (±62.98) a	199.12 (±175.95) a

**Table 4:** Correlation results among all the tested parameters in assay II, using seedlings.

ASSAY II USING SEEDLINGS							End-Point PCRs		Real-Time PCRs		Virus Copy Number
		Donor Number	Virus Strain (0 None, 1 E-5, 2 L-18)	Lesion Area (mm^2^)	Original Virus Concentration (ng/µL)	VCG Compatibility (0 None, 1 Yes)	Qiagen Extract (ng/µL)	Takara Dye Toothpick (ng/µL)	Takara III Extract (ng/µL)	Takara III Toothpick (ng/µL)	
Donor number	Pearson Correlation	1									
	Sig. (2-tailed)										
Virus strain (0 None, 1 E-5, 2 L-18)	Pearson Correlation	−0.255	1								
	Sig. (2-tailed)	0.042									
Lesion area (mm^2^)	Pearson Correlation	0.579	−0.635	1							
	Sig. (2-tailed)	5.277E-7	1.785E-8								
Original virus concentration (ng/µL)	Pearson Correlation	−0.768	0.701	−0.779	1						
	Sig. (2-tailed)	1.238E-13	1.081E-10	3.427E-14							
VCG compatibility (0 None, 1 Yes)	Pearson Correlation	−0.716	0.832	−0.793	0.963	1					
	Sig. (2-tailed)	3.010E-11	1.631E-17	5.926E-15	6.315E-37						
Qiagen Extract (ng/µL)	Pearson Correlation	−0.633	0.690	−0.766	0.829	0.852	1				
	Sig. (2-tailed)	1.936E-8	2.796E-10	1.706E-13	2.564E-17	4.482E-19					
Takara Dye Toothpick (ng/µL)	Pearson Correlation	−0.634	0.690	−0.766	0.829	0.852	1.000	1			
	Sig. (2-tailed)	1.925E-8	2.817E-10	1.700E-13	2.535E-17	4.447E-19	1.64E-159				
Takara III Extract (ng/µL)	Pearson Correlation	−0.635	0.689	−0.765	0.830	0.852	1.000	1.000	1		
	Sig. (2-tailed)	1.721E-8	3.192E-10	1.773E-13	2.469E-17	4.779E-19	4.645E-129	3.422E-127			
Takara III Toothpick (ng/µL)	Pearson Correlation	−0.633	0.690	−0.766	0.829	0.852	1.000	1.000	1.000	1	
	Sig. (2-tailed)	1.957E-8	2.800E-10	1.707E-13	2.589E-17	4.488E-19	3.336E-154	1.198E-167	8.542E-125		
Virus copy number	Pearson Correlation	−0.271	0.575	−0.543	0.535	0.583	0.775	0.774	0.773	0.775	1
	Sig. (2-tailed)	0.030	6.540E-7	3.644E-6	5.281E-6	4.220E-7	5.990E-14	6.310E-14	7.26E-14	6.015E-14	
	Negative correlation is significant at the 0.05 level (2-tailed).										
	Positive correlation is significant at the 0.05 level (2-tailed).										
	N	64	64	64	64	64	64	64	64	64	64

**Table 5:** Correlation results among all the tested parameters in assay II, using branches.

ASSAY II USINGBRANCHES							End-Point PCRs		Real-Time PCRs		Virus Copy Number
		Donor Number	Virus Strain (0 None, 1 E-5, 2 L-18)	Lesion Area (mm^2^)	Original Virus Concentration (ng/µL)	VCG Compatibility (0 None, 1 Yes, 2 No)	Qiagen Extract (ng/µL)	Takara Dye Toothpick (ng/µL)	Takara III Extract (ng/µL)	Takara III Toothpick (ng/µL)	
Donor number	Pearson Correlation	1									
	Sig. (2-tailed)	1									
Virus strain (0 None, 1 E-5, 2 L-18)	Pearson Correlation	6.74337E-18	1								
	Sig. (2-tailed)	1									
Lesion area (mm^2^)	Pearson Correlation	0.203	−0.032	1							
	Sig. (2-tailed)	1.562E-2	0.706								
Original virus concentration (ng/µL)	Pearson Correlation	−0.238	0.897	−0.096	1						
	Sig. (2-tailed)	5E-3	1E-4	0.257							
VCG compatibility (0 None, 1 Yes, 2 No)	Pearson Correlation	−0.459	0.562	0.156	0.675	1					
	Sig. (2-tailed)	1.14525E-08	4.73908E-13	6.539E-2	1E-4						
Qiagen Extract (ng/µL)	Pearson Correlation	−0.199	−0.018	−0.871	0.072	−0.134	1				
	Sig. (2-tailed)	1.789E-2	0.828	1.65686E-44	0.395	0.113					
Takara Dye Toothpick (ng/µL)	Pearson Correlation	−0.199	−0.018	−0.871	0.072	−0.134	0.999	1			
	Sig. (2-tailed)	1.788E-2	0.828	1.64435E-44	0.395	0.113	1E-4				
Takara III Extract (ng/µL)	Pearson Correlation	−0.199	−0.018	−0.871	0.072	−0.134	0.999	0.999	1		
	Sig. (2-tailed)	1.788E-2	0.828	1.73168E-44	0.395	0.113	1E-4	1E-4			
Takara III Toothpick (ng/µL)	Pearson Correlation	−0.199	−0.018	−0.871	0.072	−0.134	0.999	0.999	0.999	1	
	Sig. (2-tailed)	1.788E-2	0.827	1.74169E-44	0.395	0.113	1E-4	1E-4	1E-4		
Virus copy number	Pearson Correlation	−0.129	−0.051	−0.383	0.004	−0.056	0.659	0.659	0.659	0.659	1
	Sig. (2-tailed)	0.128	0.549	1E-4	0.964	0.510	8.397E-19	8.639E-19	8.326E-19	8.519E-19	
	Negative correlation is significant at the 0.05 level (2-tailed).										
	Positive correlation is significant at the 0.05 level (2-tailed).										
	N	140	140	140	140	140	140	140	140	140	140

## Data Availability

Not applicable.

## Supplementary Materials

The following supporting information can be downloaded at: https://www.mdpi.com/article/10.3390/v14122678/s1, Table S1. Correlation results among all the tested parameters in assay I, using seedlings. Table S2 Correlation results among all the tested parametersin assay I, using branches.
