# Supplementary material for: Canker Development and Biocontrol Potential of CHV-1 Infected English Isolates of Cryphonectria parasitica Is Dependent on the Virus Concentration and the Compatibility of the Fungal Inoculums
Source: Viruses. 2022 Nov 29;14(12):2678. doi: 10.3390/v14122678 (PMC9785502; doi:10.3390/v14122678)
Supplement: Supplementary file 1 [file viruses-14-02678-s001.zip › viruses-2027050-supplementary.pdf]

Supplementary Table S1. Correlation results among all the tested parameters in assay I, using seedlings.

ASSAY I USING SEEDLINGS						End-point PCRs		Real-time PCRs		Virus copy number
		Inoculum number	Virus strain (0 None, 1 E-5, 2 L-18)	Lesion area (mm2)	Original virus concentration (ng/ul)	Qiagen Extract (ng/ul)	Takara Dye Toothpick (ng/ul)	Takara III Extract (ng/ul)	Takara III Toothpick (ng/ul)	
Inoculum number	Pearson Correlation	1								
	Sig. (2-tailed)									
Virus strain (0 None, 1 E-5, 2 L-18)	Pearson Correlation	-0.621	1							
	Sig. (2-tailed)	6.852E-6								
Lesion area (mm2)	Pearson Correlation	0.754	-0.628	1						
	Sig. (2-tailed)	3.519E-9	4.928E-6							
Original virus concentration (ng/ul)	Pearson Correlation	-0.882	0.818	-0.738	1					
	Sig. (2-tailed)	2.587E-15	1.270E-11	1.084E-8						
Qiagen Extract (ng/ul)	Pearson Correlation	-0.801	0.657	-0.695	0.854	1				
	Sig. (2-tailed)	6.318E-11	1.243E-6	1.659E-7	1.701E-13					
Takara Dye Toothpick (ng/ul)	Pearson Correlation	-0.802	0.657	-0.695	0.854	1.000	1			
	Sig. (2-tailed)	6.111E-11	1.251E-6	1.657E-7	1.705E-13	3.355E-106				
Takara III Extract (ng/ul)	Pearson Correlation	-0.802	0.658	-0.695	0.854	1.000	1.000	1		
	Sig. (2-tailed)	6.281E-11	1.239E-6	1.659E-7	1.743E-13	1.6E-103	5.19E-112			
Takara III Toothpick (ng/ul)	Pearson Correlation	-0.774	0.636	-0.665	0.832	0.984	0.984	0.984	1	
	Sig. (2-tailed)	6.248E-11	1.264E-6	1.672E-7	1.760E-13	5.289E-111	2.181E-119	1.383E-112		
Virus copy number	Pearson Correlation	-0.205	0.325	-0.227	0.247	0.510	0.510	0.510	0.507	1
	Sig. (2-tailed)	0.181	0.031	0.138	0.107	0.0004	0.0004	0.0003	0.0004	
(dark shading)	Negative correlation is significant at the 0.05 level (2-tailed).									
(light shading)	Positive correlation is significant at the 0.05 level (2-tailed).									
	N	44	44	44	44	44	44	44	44	44

Supplementary Table S2. Correlation results among all the tested parameters in assay I, using branches.

ASSAY I USING BRANCHES						End-point PCRs		Real-time PCRs		Virus copy number
		**Inoculum number**	Virus strain (0 None, 1 E-5, 2 L-18)	Lesion area (mm2)	Original virus concentration (ng/ul)	Qiagen Extract (ng/ul)	Takara Dye Toothpick (ng/ul)	Takara III Extract (ng/ul)	Takara III Toothpick (ng/ul)	
Inoculum number	Pearson Correlation	1								
	Sig. (2-tailed)									
Virus strain (0 None, 1 E-5, 2 L-18)	Pearson Correlation	-0.621059003	1							
	Sig. (2-tailed)	1.149E-4								
Lesion area (mm2)	Pearson Correlation	0.74075626	-0.573329364	1						
	Sig. (2-tailed)	8.25551E-07	4.872E-4							
Original virus concentration (ng/ul)	Pearson Correlation	-0.763085723	0.947139243	-0.618621356	1					
	Sig. (2-tailed)	2.42429E-07	7.39719E-17	1.244E-4						
Qiagen Extract (ng/ul)	Pearson Correlation	-0.631351624	0.701417699	-0.536603299	0.808636403	1				
	Sig. (2-tailed)	8.15436E-05	5.43413E-06	1.285E-3	1.24752E-08					
Takara Dye Toothpick (ng/ul)	Pearson Correlation	-0.677587885	0.764437702	-0.567765792	0.850110249	0.946747663	1			
	Sig. (2-tailed)	1.4805E-05	2.24142E-07	5.684E-4	3.84052E-10	8.27108E-17				
Takara III Extract (ng/ul)	Pearson Correlation	-0.694589536	0.658301711	-0.547004191	0.81279874	0.952502458	0.936502481	1		
	Sig. (2-tailed)	7.3123E-06	3.12419E-05	9.873E-4	9.15058E-09	1.46261E-17	1.17742E-15			
Takara III Toothpick (ng/ul)	Pearson Correlation	-0.765735589	0.798113709	-0.59992026	0.907321694	0.927070585	0.945964974	0.943959032	1	
	Sig. (2-tailed)	2.07785E-07	2.64421E-08	2.290E-4	3.36468E-13	9.42806E-15	1.03136E-16	1.78935E-16		
Virus copy number	Pearson Correlation	-0.33596088	0.10597614	-0.1840679	0.26755686	0.51294676	0.43727226	0.51182069	0.54341257	1
	Sig. (2-tailed)	0.05593902	0.5572241	0.30516821	0.13223907	2.269E-3	1.093E-2	2.329E-3	1.082E-3	
	Negative correlation is significant at the 0.05 level (2-tailed).									
	Positive correlation is significant at the 0.05 level (2-tailed).									
	N	33	33	33	33	33	33	33	33	33
